# Side-Slither Data-Based Vignetting Correction of High-Resolution Spaceborne Camera with Optical Focal Plane Assembly

**DOI:** 10.3390/s18103402

**Published:** 2018-10-11

**Authors:** Chaochao Chen, Jun Pan, Mi Wang, Ying Zhu

**Affiliations:** State Key Laboratory of Information Engineering in Surveying, Mapping and Remote Sensing, Wuhan University, Wuhan 430079, China; cygeek@whu.edu.cn (C.C.); wangmi@whu.edu.cn (M.W.); yzhu1003@whu.edu.cn (Y.Z.)

**Keywords:** optical focal plane assembly, vignetting correction, push-broom, high-resolution optical satellite, side-slither data, power-law model, gray level co-occurrence matrix, Levenberg–Marquardt

## Abstract

Optical focal plane assemblies are increasingly being used in high-resolution optical satellite systems to enhance the width of the image using linear push-broom imaging. With this system, vignetting occurs in the area of overlap, affecting image quality. In this paper, using the characteristics of the side-slither data, we propose side-slither data-based vignetting correction of a high-resolution spaceborne camera with an optical focal plane assembly. First, the raw side-slither data standardization is used to ensure that each row has the same features. Then, with the spatial correlation of a gray-level co-occurrence matrix, the gray-level co-occurrence matrix is proposed to identify the uniform regions, to extract the sample points. Finally, due to the characteristics of compatible linear response and non-linear response, the power-law model was used to fit, and the Levenberg–Marquardt algorithm was used to fit the model. In the experiment, polynomial fitting, laboratory coefficients and on-orbit coefficients were used for comparison with the proposed method. The side-slither data can be treated as a uniform scene due to their characteristics, and the side-slither image that was corrected using the proposed method showed less than 1% change in mean value, a root-mean-square deviation value better than 0.1%, and the average streaking metrics were superior to 0.02. The results showed that the proposed method performs significantly better in the vignetting area.

## 1. Introduction

**Figure 1:**
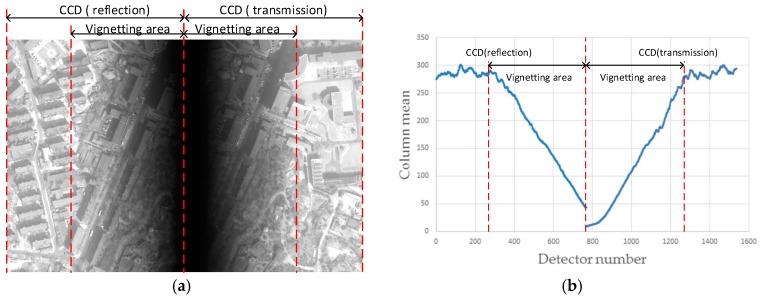
The impact of vignetting on image quality. (**a**) The raw data using the optical butting system; (**b**) the column mean of the raw data show in (**a**).

The use of high-resolution optical satellite imagery has increased over the past decade, reaching the highest level at the decimeter level and moving toward the centimeter level. The push-broom camera has attracted a great deal of attention due to its high signal-to-noise ratio (SNR). To extend the width of high-resolution optical remote-sensing satellites, charge coupled device (CCD) focal plane assemblies have been used to meet the requirement for a large field of view (FOV) [1,2,3,4]. At present, mainly mechanical butting and optical butting are used. Mechanical butting usually involves a few CCDs arranged in a straight line or a two-row staggered assembly in a focal plane. Optical butting can be divided into refractive, transflective and reflective types. Among these, refractive butting produces aberrations, and the energy utilization of transflective butting is less than 50%. Reflective butting has several advantages, including greater butting length, high energy efficiency, low weight and the lack of aberration. However, reflective butting results in vignetting in areas of overlap, the elimination of which requires image processing [5]. Figure 1 shows the impact of vignetting on image quality. The left image of Figure 1a represents the reflection CCD, and the right image represents the transmission CCD, with the vignetting area in the middle, as can be seen from Figure 1a. Due to the impact of vignetting, the features of the image in the vignetting area cannot be identified, which will be hard to use. Figure 1b shows the column mean of the image of Figure 1a. It shows that the brightness value of the image in the vignetting area is degraded, which greatly affects the interpretation of the image. Therefore, for the optical butting imaging system, it is essential to do the vignetting correction efficiently and accurately.

To eliminate the vignetting in the linear push-broom imaging with the optical butting system, traditional vignetting correction methods are the look-up table method, progressive scan method and vignetting approximation method [6,7,8,9,10,11]. The simple look-up method and the vignetting approximation method require reference images for calibration, such as laboratory images and multi-luminance images. The simple look-up requires a standard image to obtain the look up table (LUT) [12]. The vignetting approximation method involves the determination of a compensation factor for each pixel under each illuminance through multiple luminances and then fitting of the function of illuminance and the compensation factor for each pixel according to this function [13]. The progressive scan method does not require camera calibration and uses the actual image data obtained by line-by-line fitting. This method is computationally intensive, and when the gray changes in two adjacent lines are large, the restored image shows striping. For the linear push-broom imaging system that uses the optical butting system, the vignetting and the pixel response non-uniformity need to be taken into account. The vignetting method based on relative radiometric calibration has been proposed for linear push-broom systems [14]. Laboratory radiometric calibration and on-orbit relative radiometric calibration are important methods of this type for remote sensing camera [15,16,17]. The characteristics of the satellite must be measured prior to launch using a laboratory integrating sphere [18]. The responses of sensors change on-orbit due to environmental variation, particulate contamination and other factors. A uniform scene is more difficult to obtain with on-orbit relative radiometric calibration, which limits the accuracy of on-orbit calibration [15].

**Figure 2:**
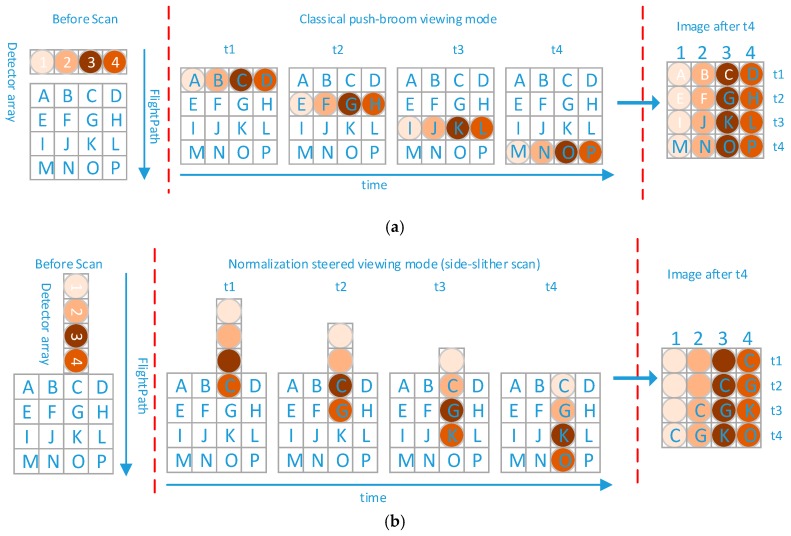
(**a**) Classical push-broom viewing mode; (**b**) normalization-steered viewing mode (side-slither scan).

With the improvement of satellite technology [19], the use of satellite yaw 90° imaging for calibration has been proposed, e.g., with QuickBird [20], RapidEye [21], Landsat 8 [22] and Pleiades-HR [23]. Satellites using optical butting, such as GaoFen-1 and GaoFen-2 [19,24], provide new vignetting calibration data by using side-slither scans. Figure 2 shows the classical push-broom viewing mode and the side-slither scan. The side-slither scan has been applied to a satellite that can rotate its array 90 degrees on the yaw axis, which means that every detector is ideally aligned to image the same target as it moves along the velocity vector. Each detector has the same feature, and information from the same feature can be collected as a uniform scene. The side-slither data are mainly used for the relative radiometric calibration. At present, it can be divided into two major calibration methods, single point calibration and using histogram to calibration. Single point calibration is mainly used for the mechanical butting, that all of the CCDs cannot image one region. It needs to take an image on the desert scene [20,21,25]. The histogram to calibration mainly uses the histogram matching [19,23]. For the optical butting system, the histograms in the non-vignetting area and vignetting area are much different.

With the characteristic of the optical butting system and the side-slither data, this paper proposes the multi-point calibration method based on the power-law model, using the spatial correlation of the gray level co-occurrence matrices to obtain the sample points. It describes a proposed side-slither data-based vignetting correction. The proposed method can be divided into three main steps: the raw side-slither data standardization based on the linear features, extracting the sample points using the gray-level co-occurrence matrices (GLCMs) and obtaining the coefficients based on the power-law model. The raw side-slither data standardization was used to ensure that each row has the same feature, and the line detection was used to ensure the accuracy. GLCMs and inverse different moment (IDM) were used to identify regions of uniformity. GLCMs describe texture by examining the spatial correlations of gray areas in the column direction. Given the characteristics of the GLCM, the sample points were confirmed through the calculation of the GLCM and IDM using the standard data. Finally, the Levenberg–Marquardt (LM) algorithm was used to calculate the correction coefficients with the power-law model, and the power-law model can be compatible with the characteristics of linear models and nonlinear models. Two sets of data were used in experimental verification; one was obtained using the side-slither scan, and the other was obtained using the classical normal push-broom mode. Side-slither data can be treated as a uniform scene in the full FOV, and therefore can be used to verify global accuracy and the effects of vignetting correction. The images acquired in the classical normal push-broom mode were used to verify the correction effects of the coefficients on different scenes. Polynomial fitting, laboratory coefficients and on-orbit coefficients were used for comparison with the proposed method. The experimental results for the side-slither data included root-mean-square deviation (RA) values exceeding 0.1%, and the averages of streaking metrics were superior to 0.02. The results of qualitative analysis and quantitative evaluation indicated that the proposed method performed better than the classical methods.

This paper is organized as follows. Section 2 presents the analysis of the vignetting on the optical butting system, which is based on reflectors. Details of the proposed method based on the power-law model are provided in Section 3. Section 4 describes image correction using the coefficients. In Section 5, the pre-processing and experimental results are described in detail. Finally, the conclusions drawn from this study are provided in Section 6.

## 2. Analysis of the Vignetting

**Figure 3:**
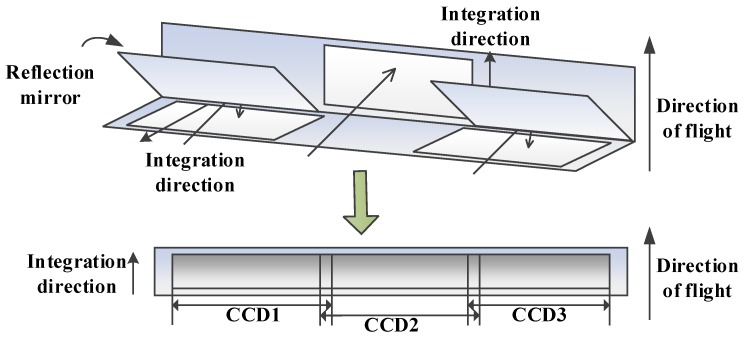
Focal plane of optical butting based on reflectors.

The optical butting system based on reflectors is characterized by high energy efficiency and collinear time delay integral (TDI)-CCD imaging. Figure 3 shows the focal plane of optical butting based on reflectors. The sensors consist of three CCDs installed in the transmission and reflection areas in the focal plane, forming a continuous virtual CCD array in an approximately straight line. However, this arrangement produces an area of overlap affected by vignetting [26].

**Figure 4:**
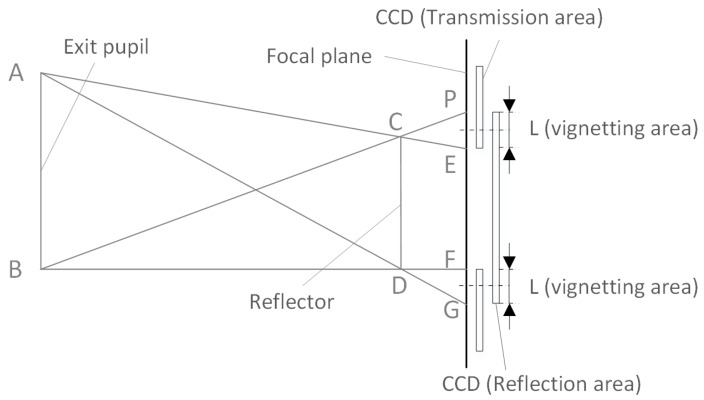
Vignetting in the focal plane-based optical butting [27].

The reflective film forms a light barrier in the transmission area, and the transmission area of the non-reflective film forms a light barrier in the reflection area. The TDI-CCD illumination on the focal surface does not change abruptly from full illumination to full light blocking; rather, it is gradual. Figure 4 shows the vignetting in the focal plane-based optical butting system.

For optical butting based on a reflector, the reflector mirror is usually implemented between the exit pupil and the focal plane of the optical system. The focal plane optical butting model is shown in Figure 4. In Figure 4, AB is the exit pupil diameter of the optical system, and CD is the length of the butting mirror. The light filled with the exit pupil is cut by the reflector mirror, and the vignetting areas PE and FG are formed on the focal plane (i.e., the CCD photosensitive surface), while the length of the CCD overlap area is L [27].

## 3. Methods

The proposed method can be divided into three steps. The side-slither data standardization using the linear features ensures that each row has the same feature, and thus provides the correct input for subsequent processing. The GLCMs and IDMs are used to identify regions of uniformity, and sample data points are derived from these regions. Finally, the LM algorithm is used to obtain the vignetting correction coefficients by fitting the power-law model.

**Figure 5:**
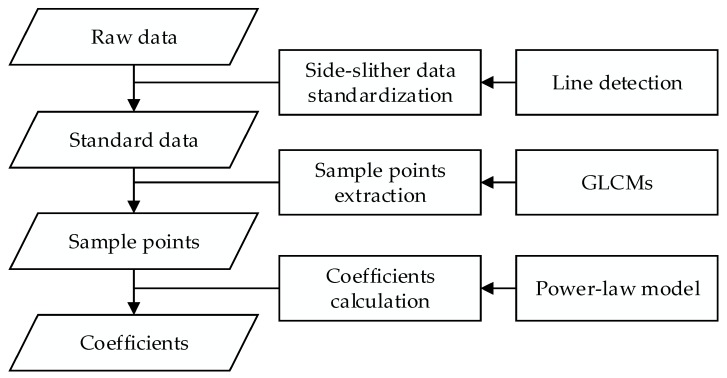
Proposed method based on the power-law model using side-slither data.

Figure 5 shows the proposed method based on the power-law model using side-slither data. The power-law model is used to fit the coefficients because of its compatibility with linear and nonlinear models. In the standardization of raw side-slither data, this paper uses the line detection to ensure that the same feature is on one line, and the standardization is performed only to move the locations of the detector values without changing the original digital number (DN) value. To fit the model, the uniform regions of different illumination are identified by using the characteristics of directional texture, and GLCMs are used to detect the directional texture. The sample points are derived from the uniform regions of different illumination. Finally, the sample points are fitted by the Levenberg–Marquardt (LM) method by using the power-law model.

### 3.1. Side-Slither Data Standardization Based on the Linear Features

To ensure the accuracy of subsequent processing, the raw side-slither data standardization based on the linear features was performed to ensure that each line had the same feature. The important process of obtaining highly accurate coefficients [25] can be divided into two main steps: basic adjustment and enhanced adjustment.

**Figure 6:**
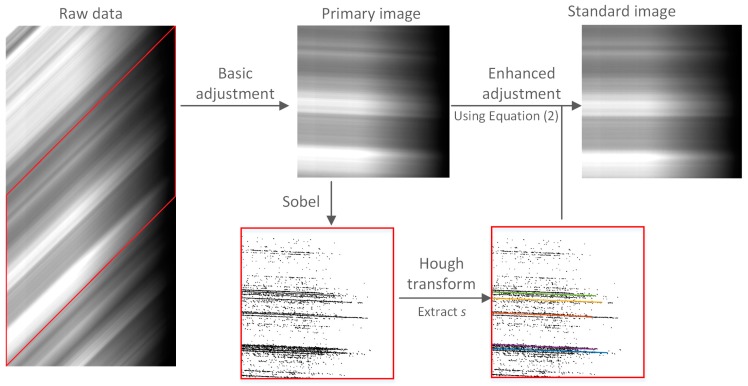
The side-slither data standardization based on the linear features.

Figure 6 describes the standardization process. Vignetting of the image is clearly visible. The detected line does not run through the entire resulting image due to the influence of the area showing vignetting. The primary image is the result of basic adjustment of the raw data, and the standard image is the result of enhanced adjustment of the primary image. In this study, the standardization process was used for further correction. First, basic adjustment was applied to the raw data. Second, the Sobel operator was used to detect the edge of the line, and Hough transform was used to determine the slope (*k*) of the line. Finally, the slope (*k*) was used for enhanced adjustment of the primary image.

Basic adjustment was based on Equation (1):

(1) $$DN_{ij}=DN_{kj},\;k=i+n-j-1,\;i=0,\;1,\;\ldots ,\;m-n-1,\;j=0,\;1,\;\ldots ,\;n-1$$

where $DN_{ij}$ is the pixel value for the primary corrected image, $DN_{kj}$ is the pixel value for the raw side-slither data, m is the total number of rows in the raw side-slither data and n is the total number of columns in the raw side-slither data.

The same feature is not at an angle of exactly 45° in the side-slither data, probably because the focal plane read-out clock cycle was out of sync when the array was oriented in the direction of motion and/or a perfect 90° side-slither angle was not achieved [25]. Therefore, enhanced adjustment was required. In the ideal case, each line of the primary image has the same feature. Figure 6 shows that the ideal state was not achieved. Therefore, further correction of the primary image was needed. In this paper, the line detection algorithm was used for further correction. First, a Gaussian filter was applied to the primary image to improve the SNR [28]. Second, the Sobel operator was used to detect lines in the image [29]. Finally, the short lines were removed, and the slopes of the remaining lines were calculated and used for enhanced adjustment of the primary image.

Figure 6 shows the resulting image after Gaussian filtering, Sobel filtering, binarization procedures and Hough transform [30]. The binarization procedures convert the detected image into a binary image with the pixels on straight lines set to 1, and the rest of the pixels set to 0. The line detection using the Hough transform is to remove discrete points and extract the slope of the line (*k*). The algorithm can detect straight lines and calculate their actual slopes after the removal of short straight lines. Figure 4d shows an image after raw data standardization:

(2) $$DN_{ij}=DN_{kj},\;k=i+\mathrm{INT}\left(s*j\right),$$

where i=0, 1, …, m−INT(s∗n)−1, j=0, 1, …, n−1 and s represents the slope of the line in Figure 6. Note that during the process of standardization, only integer shifts are applied to avoid data resampling.

### 3.2. Sample Points Extraction Using the GLCM

To determine the response characteristics of the detectors in the vignetting area for different radiances, uniform regions that respond to different radiances must be identified. However, searching for uniform regions in the vignetting area is difficult. Therefore, data from non-vignetting areas are used to determine the uniformity of the area based on the characteristic of the side-slither data that each line has the same feature. Considering the effect of pixel response non-uniformity, the GLCM was used to determine the uniformity region because of the direction texture of GLCM.

**Figure 7:**
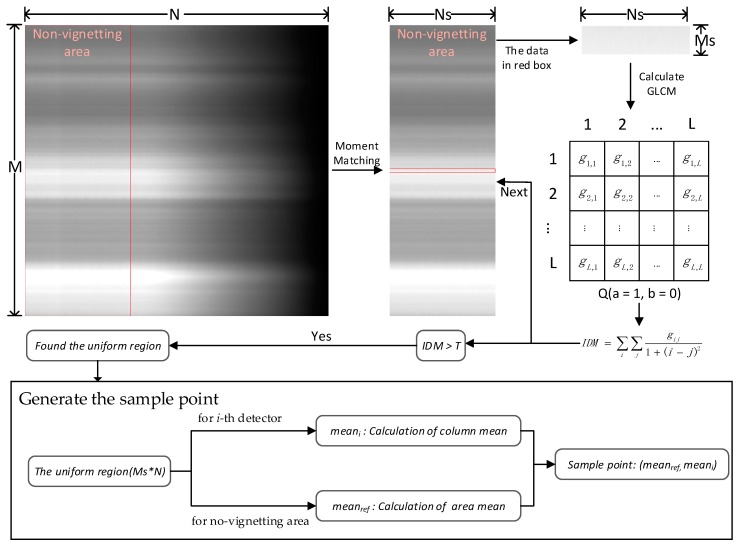
Process of the extraction of the sample points using the GLCM. IDM, inverse different moment.

Figure 7 shows the process of the extraction of the sample points using the GLCM, which is divided into three steps: moment matching, uniformity region determination and generation of sample data. Here, the size of the standard image is assumed to be M∗N, where the size of the non-vignetting area is M∗Ns. First, the non-vignetting area in the standard data was determined, and moment matching was applied in this area to remove the influence of noise. Then, the area of size *Ms* ∗ *Ns* in the non-vignetting area was chosen by the order of rows to determine whether the area was uniform. The GLCM for this area was calculated, and the IDM was obtained. If the IDM met the threshold *T*, this area was regarded as uniform, and the start line of the area was recorded; otherwise, the next area was chosen. Finally, the column mean value for the chosen area, of size *Ms* ∗ *N* on the standard image, was calculated.

In the push-broom imaging mode, the satellite image noise is mainly streaking noise. Therefore, before the uniform area is determined, the streaking noise needs to be removed. Moment matching [31] employs the streaking noise removal algorithm based on the spatial domain. It assumes that the features detected by each detector have the same irradiance distribution. Then, the characteristics of the GLCM are used to identify regions of uniformity [32]. We require only that the region be uniform in the column direction, i.e., within data from a single detector. In addition, the GLCM describes the texture by studying the spatial correlation of grayscale areas. Therefore, it can be used to analyze the texture of a certain direction to determine the uniform region.

Textures do not contain information about the relative positions of pixels calculated using only the histogram. This information is important when describing the texture, and one way to incorporate it into texture analysis is to consider not only the distribution of intensities, but also the relative positions of pixels in an image. Let *Q* = (*a*, *b*) be an operator that defines the positions of two pixels relative to each other, and consider an image f, with L possible intensity levels. Let *G* be a matrix whose element $g_{ij}$ is the number of times that pixel pairs with intensities $z_{i}$ and $z_{j}$ occur in f in the position specified by *Q*, where 1≤i,j≤L. A matrix formed in this manner is referred to as a GLCM. When the meaning is clear, the GLCM is referred to simply as *G*.

Here, we were concerned about uniformity in the column direction, so *Q* = (1, 0) was chosen to construct the GLCM. Given the characteristics of the GLCM, the concentration of values on its diagonal increases with image uniformity. The IDM reflects the uniformity of image texture and measures how much this texture changes locally. A large value indicates a lack of change in image texture and a high degree of local uniformity. The formula for the IDM is:

(3) $$\;IDM=\;\underset{i}{\sum }\underset{j}{\sum }\frac{g_{ij}}{1+{\left(i-j\right)}^{2}},\;i,j=0,1,\ldots ,L-1\;$$

where $g_{ij}$ is the GLCM value, and *L* is the intensity level. In this study, the threshold *T* was set to filter out the uniform area; if the *IDM* is greater than *T*, the area is considered to be uniform.

To fit the model, it is necessary to acquire sample points according to the uniform scenes. Therefore, for each detector, sampling points of different brightness will be introduced next. The column mean value for the uniform area, of the size *Ms* ∗ *N* on the standard image, was calculated as:

(4) $$\;mean_{i}=\frac{1}{Ms}*\underset{j}{\sum }DN_{ji},\;i=0,1,\ldots ,N-1,j=0,1,\ldots ,Ms\;$$

where $mean_{i}$ is the mean value from the i-th detector and $DN_{ji}$ is the value for the image. Since the non-vignetting area is not affected by vignetting, we use the mean of the non-vignetting area (*Ms* ∗ *Ns*) as the reference value. For this area, the reference value was described as:

(5) $$\;mean_{ref}=\frac{1}{Ns}*\underset{i}{\sum }mean_{i},\;i=0,1,\ldots ,Ns-1\;$$

where $mean_{i}$ is the mean value from the i-th detector and $mean_{ref}$ is the reference value for this area. The sample data ($mean_{ref}$, $\;mean_{i}$) from the i-th detector were recorded.

### 3.3. Coefficients Calculation Based on the Power-Law Model

In Section 3.2, the sample points have been determined. The coefficients are calculated in this section. The parameters of the power-law model can be fitted with these sample points.

**Figure 8:**
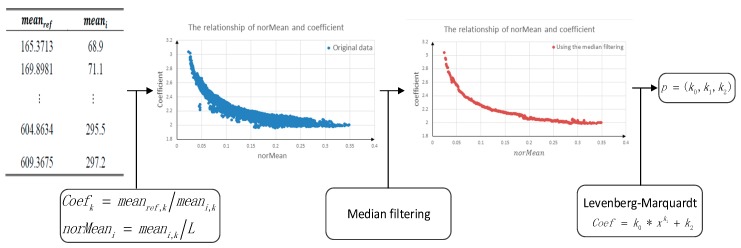
Obtain the coefficients based on the power-law model.

Figure 8 shows the process used to obtain the parameters of $p=\left(k_{0},k_{1},k_{2}\right)$, which was divided into three steps. First, the sample point data were normalized, and the sample coefficients were calculated. Second, to maintain fitting accuracy, median filtering was used. Finally, the fitting parameters were obtained using the LM algorithm to fit the model.

**Figure 9:**
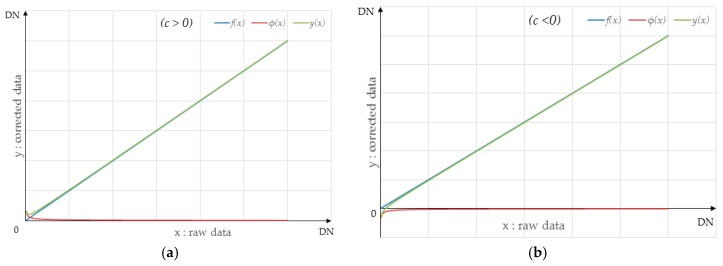
Simulation of the correction model with φ(x) set as the power-law function for: (**a**) *c* > 0; (**b**) *c* < 0.

According to the radiometric response, radiometric correction can be divided into methods using a linear model, such as QuickBird [20], and those using a nonlinear model, such as Pleiades-HR [23]. Considering the characteristics of these methods, the correction model of each detector can be divided into two parts: the linear transformation f(x) in the system and the irregular disturbance φ(x) in the system. If the response is linear, φ(x) should be constant. The correction model for the *i*-th detector can be described as:

(6) $$\{\begin{array}{c} y\left(x\right)=f\left(x\right)+\varphi \left(x\right)\\ f\left(x\right)=k*x \end{array}$$

where x is the pixel value before correction of the *i*-th detector, for which the dark current has been removed on-orbit, and y is the corrected pixel value of the *i*-th detector. In the vignetting area, radiance is concentrated mainly in the region of low luminance. The response of the detector in the low-luminance area may not be the same as those in brighter areas. The choice of φ(x) should reflect this difference. The power-law function has a long tail effect, i.e., it has a large change in a certain area, but tends to be stable in most areas. To be compatible with the linear and nonlinear conditions in the low-luminance region, this paper proposes the function of φ(x) as a power-law model. Therefore, φ(x) is described in this study using the power-law function:

(7) $$\varphi \left(x\right)=c*x^{-r},\;r\geq 0$$

where c and r are the function parameters. Figure 9 shows a simulation of the correction model, with φ(x) set as a power-law function.

The blue line represents the linear transformation f(x); the red line represents the power-law function φ(x); and the green line represents the correction model y(x). Figure 9a presents a simulation for *c* > 0, and Figure 9b presents a simulation for *c* < 0. Figure 9 shows the influence of the power-law function on the linear part, which is concentrated mainly in the low-luminance region. It is determined by the long tail effect of the power-law function. Therefore, the correction model for the *i*-th detector proposed in this paper can be described as:

(8) $$\;y\left(x\right)=k*x+c*x^{-r}=\left(k+c*x^{-\left(r+1\right)}\right)*x.$$

Therefore, Coef(x) can be described as:

(9) $$Coef\left(x\right)=k_{2}+k_{0}*x^{k_{1}},\;k_{0}=c,\;k_{1}=-\left(r+1\right),\;k_{2}=k\;$$

where $p=\left(k_{0},k_{1},k_{2}\right)$ is the parameter to be solved in this paper, and the correction model is:

(10) y(x)=Coef(x)∗x

**Figure 10:**
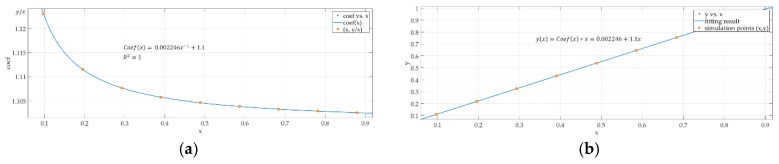
The result of fitting 10 points that are linear. (**a**) The coefficients obtained using the simulation points; (**b**) the fitting result (blue line).

When *r* = 0 in the power-law function, the correction model can be treated as a linear model. The power-law model can degenerate into a linear model if the response is linear, and Figure 10 simulates 10 points that are linear to show the performance of the power-law model when the response is linear.

Figure 10 shows the result of fitting 10 points that are linear. Figure 10a shows the coefficients obtained using the simulation points based on the power-law model. It can be seen that $k_{1}=-1$, while the response is linear. Figure 10b shows the fitting result (blue line), and the orange points represent the simulation points, The power-law model can be degenerated into a linear model while the response is linear. The results show that when the response is linear, the effect of fitting using the power-law model is consistent with that of using the linear model. Therefore, it can be seen that the power-law model can be compatible with both the linear response and the nonlinear response. Next, the paper describes the process of obtaining the coefficients using the power-law models.

After the sample points were normalized, the sample coefficients were calculated as follows:

(11) $$coef_{k}=mean_{ref,k}/mean_{i,k}$$

where *k* is the number of samples, $mean_{ref,k}$ is the reference value for the *k*-th sample data point and $mean_{i,k}$ is the value for the *k*-th sample data point from the *i*-th detector. Normalization can be described as:

(12) $$norMean_{i,k}=mean_{i,k}/L$$

where $norMean_{i,k}$ is the normalization value for the *k*-th sample data point from the *i*-th detector and *L* is the intensity level.

Then, to remove noise from the sample points, a previously described median filtering algorithm [33] was used. Coefficients were determined for each sample space using median filtering, and only one sample was reserved for each sample space. The LM algorithm [34], a well-known optimization algorithm for determining parameters of non-linear systems, was used to determine the parameters *k*, *c* and *r*.

## 4. Results

### 4.1. Experimental Data

YaogGan-26 is an optical remote-sensing satellite launched in December 2014. The focal plane of YaogGan-26 is assembled using a reflective optical butting system, which ensures that all detectors are aligned in a plane. Therefore, each detector can take an image of the same features using side-slither scanning. Due to this high-resolution remote-sensing satellite’s strong agility and attitude control capabilities, side-slither data could be obtained using the side-slither scan.

In this study, the coefficients obtained by the proposed method were used in the vignetting correction of the processing system. A total of three groups of data was used for calibration and verification: Group A, side-slither data used to calculate the correction coefficients; Group B, another set of side-slither data used to verify the coefficients; and Group C, data obtained using the classical model including water, city, hill and desert scenes, which were used to verify the effects of correction coefficients. The size of the single CCD that was cropped was 1036 × 768, and the size of the overlap area is 1036 × 500.

In this section, we present an analysis comparing results of images corrected using three methods: (1) vignetting image correction based on polynomial fitting [35]; (2) laboratory coefficients; (3) on-orbit coefficients based on a lookup table from histogram matching images between February and April 2015; (4) the proposed side-slither-based method.

Table 1 shows the details of the experimental data. Here, three groups of data were introduced: Group A was the calibration data, and Groups B and C were the verification data.

### 4.2. Accuracy Assessment for the Results from Group B

Due to the characteristics of the side-slither data, in which each row has the same feature, these data can be treated as a uniform field. To evaluate the effect of the corrected images, two indicators were used: the root-mean-square deviation (RA) and streaking metrics. The RA is a measure of image uniformity that is used mainly for homogeneous scenes. Streaking metrics can be used to evaluate streaks.

The RA is used to evaluate uniform field images and is also referred to as the relative radiometric accuracy [36]. It is calculated as follows:

(13) $$RA=\frac{\sqrt{\frac{\sum_{i=1}^{n}{\left(Mean_{i}-\bar{Mean}\right)}^{2}}{n}}}{\bar{Mean}}*100\%$$

where $Mean_{i}$ is the mean value from the i-th detector, $\bar{Mean}$ is the mean value for the image and n is the total number of columns in the image. Smaller RA values reflect greater accuracy.

The streaking metrics are sensitive to detector-to-detector non-uniformity (streaking) [22,37,38] and are therefore used for detector comparison. They can be described as follows:

(14) $$streaking_{i}=\frac{\left|Mean_{i}-\frac{1}{2}\left(Mean_{i-1}+Mean_{i+1}\right)\right|}{\frac{1}{2}\left(Mean_{i-1}+Mean_{i+1}\right)}*100$$

where $Mean_{i}$ is the mean value from the i-th detector. Lower streaking metrics values reflect greater image uniformity. In this paper, three different brightness regions of side-slither data were selected to verify the effect of the vignetting correction.

**Figure 11:**
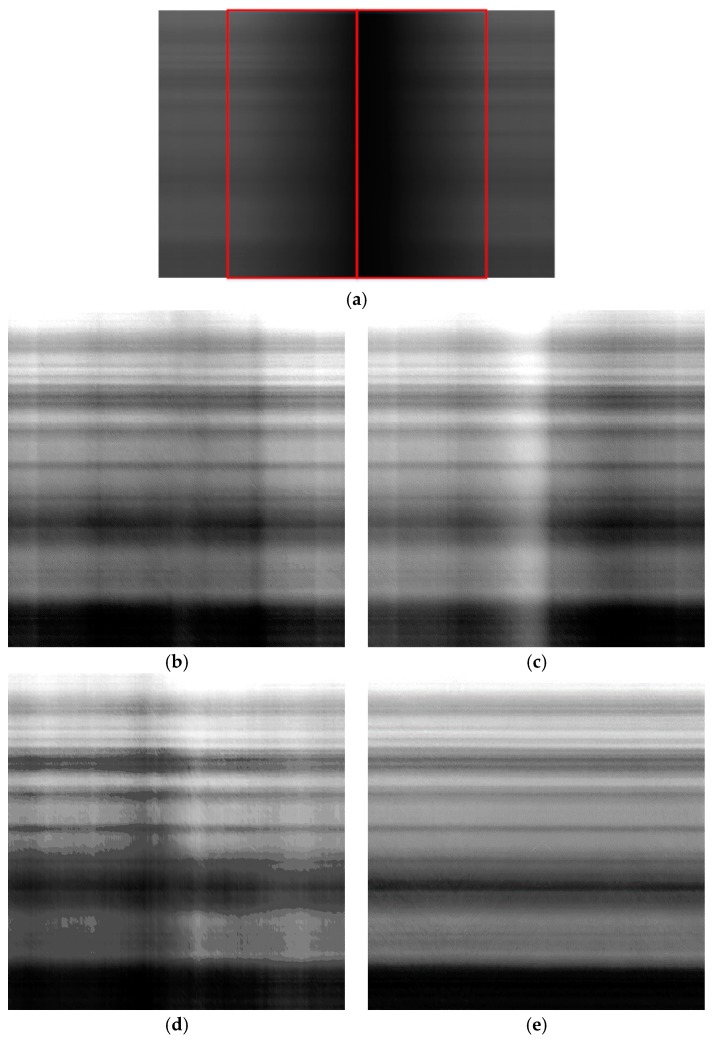
Corrected images of the low-brightness region of the side-slither data (the images were stretched for display): (**a**) raw data for the low-brightness region; (**b**) results obtained using polynomial fitting; (**c**) results obtained using laboratory coefficients; (**d**) results obtained using the on-orbit coefficients; (**e**) results generated using coefficients obtained by the proposed method.

**Figure 12:**
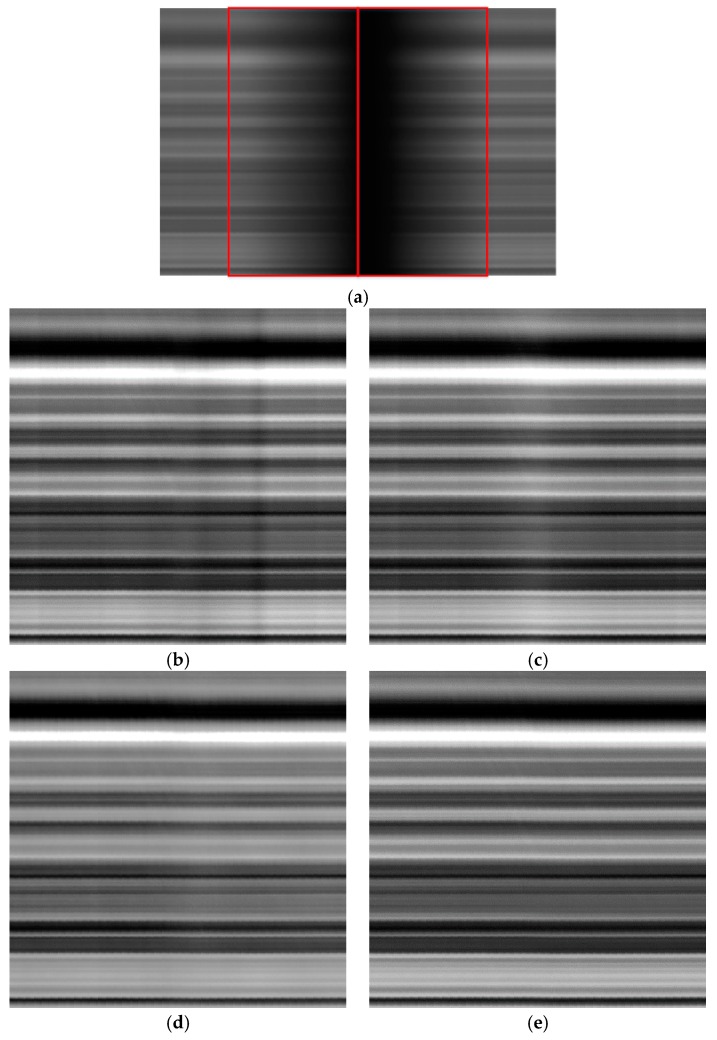
Corrected images of the middle brightness region of the side-slither data (the images were stretched for display): (**a**) raw data for the low-brightness region; (**b**) results obtained using polynomial fitting; (**c**) results obtained using laboratory coefficients; (**d**) results generated using on-orbit coefficients; (**e**) results generated using coefficients obtained by the proposed method.

**Figure 13:**
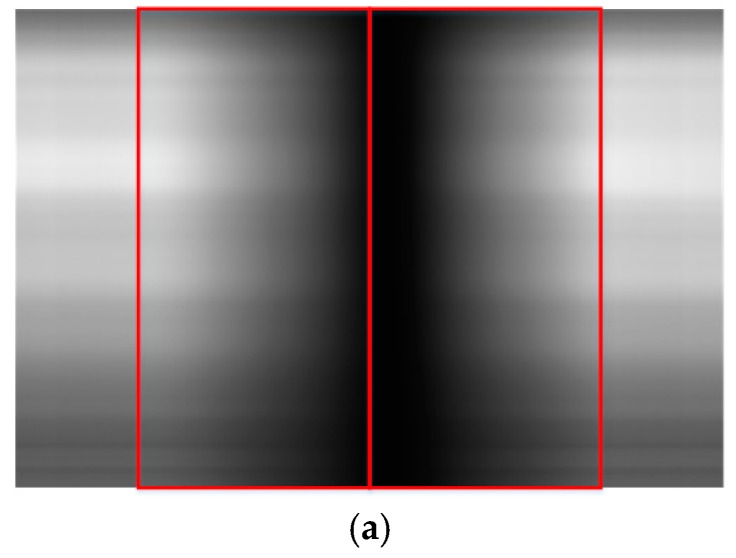

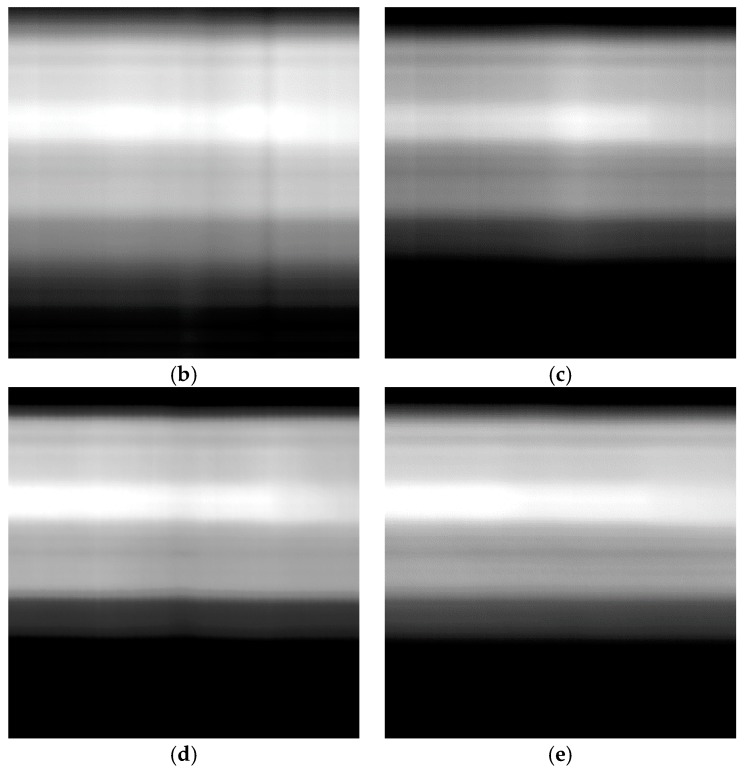
Corrected images of the high-brightness region of the side-slither data (the images were stretched for display): (**a**) raw data for the low-brightness region; (**b**) results obtained using polynomial fitting; (**c**) results obtained using laboratory coefficients; (**d**) results generated using on-orbit coefficients; (**e**) results generated using coefficients obtained by the proposed method.

Figure 11, Figure 12 and Figure 13 show the results before and after correction for the three brightness regions. Since the sensor provides data with 10 bits of digitization, the corrected images were stretched in order to better display the effects of applying the different correction methods. Figure 11 shows the results for the low-brightness region. Figure 11a shows the image before correction; the left and right sides of the red box represent the vignetting areas of the left and right CCDs, respectively. Figure 11b shows the result obtained using the polynomial fitting, this method being based on the single image. Figure 11c shows the results obtained using the laboratory coefficients with the overlapping area removed; the image size is 1036 × 1036. Figure 11d shows the results generated using the on-orbit coefficients; the image size is 1036 × 1036. Figure 11e shows the results obtained using the proposed method; the image size is 1036 × 1036. Figure 11b shows the result was non-uniform. The influence of detectors was neglected while polynomial fitting was used to calculate the vignetting function. The polynomial fitting did not guarantee a better relative radiometric correction. Therefore, Figure 11b, Figure 12b and Figure 13b show streaking. As can be seen in Figure 11c, the results obtained using the laboratory coefficients were non-uniform. Figure 11d shows a noticeable non-uniformity in the vignetting area; it shows that the on-orbit coefficients was useless in the low-brightness region. The vignetting area in Figure 11e reflects better performance than does that in Figure 11c. Figure 12 shows the images before and after correction for the middle brightness region. Figure 13 shows the results for the high-brightness area. From the perspective of continuity and uniformity, comparison of the on-orbit coefficients and the proposed method showed no marked difference (Figure 12 and Figure 13). The correction effects of laboratory coefficients in these three different regions are not satisfactory. As the side-slither data can be treated as uniform field data, to better describe the details and differences, the following analysis of column means was performed.

**Figure 14:**
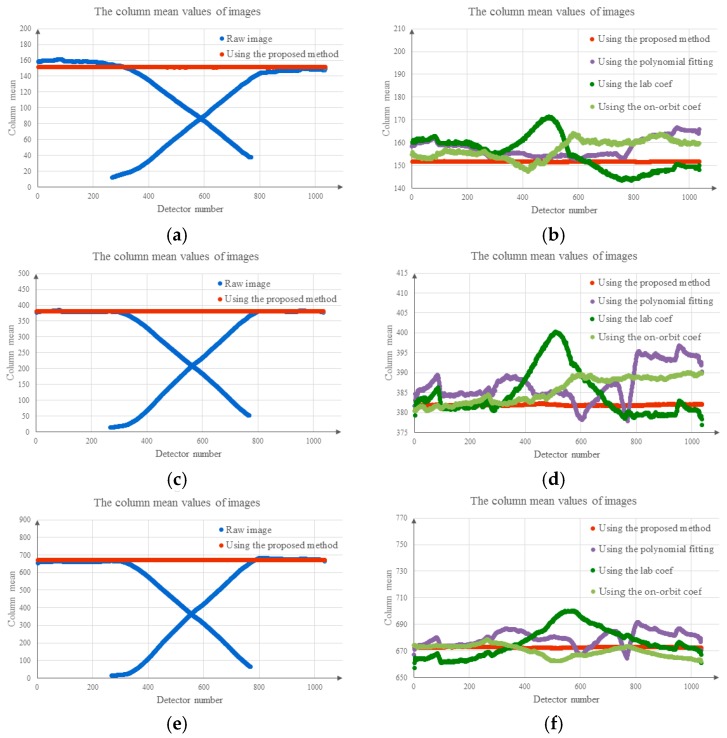
Column mean values for the raw data and corrected images: (**a**) low-brightness region, column mean values for the raw data and results obtained using the proposed method; (**b**) low-brightness region, column mean values for the corrected images; (**c**) middle brightness region, column mean values for the raw data and results obtained using the proposed method; (**d**) middle brightness region, column mean values for the corrected images; (**e**) high-brightness region, column mean values for the raw data and results obtained using the proposed method; (**f**) high-brightness region, column mean values for the corrected images.

Figure 14 shows the column means for the images before and after correction, where the abscissa represents the detector number and the ordinate represents the column mean values for the image. The blue dots represent the column means for the raw images; the dark green dots represent the column means for the images corrected using the laboratory coefficients; the red dots represent the column means for the results obtained using the proposed method; and the light green dots represent the column means for the images corrected using the on-orbit coefficients. Figure 14a,b shows the column means for the low-brightness region, corresponding to Figure 11. Figure 14c,d shows the column means for the middle brightness region, corresponding to Figure 12. Figure 14e,f shows the column means for the high-brightness region, corresponding to Figure 13. Figure 14a,c,e shows that the proposed method performed well with regard to the vignetting areas. Figure 14b,d,f shows that the column means for the vignetting and non-vignetting areas differed in the image corrected using the polynomial fitting, the laboratory coefficients and the on-orbit coefficients, whereas the image corrected using the coefficients obtained with the proposed method reflects good performance. Compared with the polynomial fitting, the laboratory coefficients and the on-orbit coefficients, the coefficients calculated by the proposed method performed well, and the corrected images were uniform. To analyze the results obtained using the different coefficients better, the distribution of streaking metrics was analyzed.

**Figure 15:**
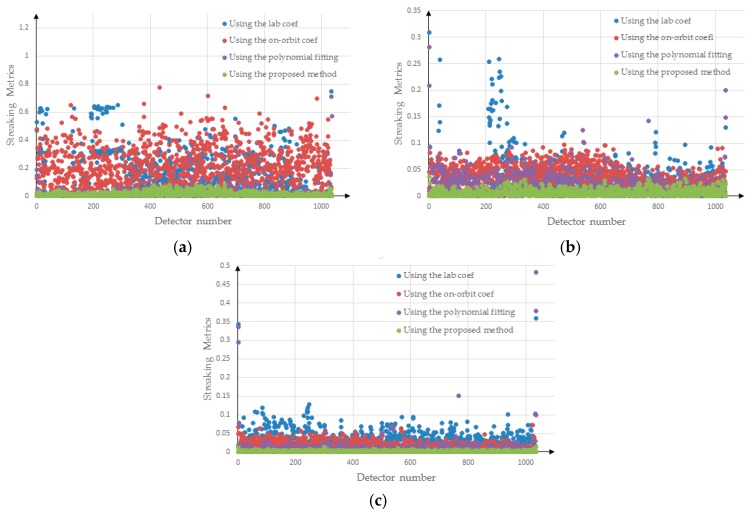
Streaking metrics for the corrected images: (**a**) low-brightness region; (**b**) middle brightness region; (**c**) high-brightness region.

Figure 15 shows the distribution of streaking metrics for the corrected images, where the abscissa indicates the detector number and the ordinate indicates the streaking metrics. The blue dots represent the streaking metrics for the images corrected using the laboratory coefficients; the red dots represent the streaking metrics for the images corrected using the method proposed in this paper; the purple dots represent the streaking metrics for the images corrected using the polynomial fitting; and the green dots represent the streaking metrics for the images corrected using the on-orbit coefficients. Figure 15a–c represents the distribution of streaking metrics for the low-brightness, middle brightness and high-brightness areas, respectively. Figure 15a shows that after correction using the laboratory coefficients, the maximum value of the streaking metrics was close to 0.8, and a large proportion of the streaking metrics for all columns had values exceeding 0.2, whereas the maximum value of the streaking metrics for the image corrected using the proposed method did not exceed 0.1. Figure 15b shows that the streaking metrics for the images corrected using the laboratory coefficients are generally less than 0.1. The streaking metrics for the images corrected using the proposed method were obviously better than those obtained using the laboratory coefficients (Figure 15b). As can be seen in Figure 15c, the method proposed in this paper was obviously better than the method based on the other methods, and the streaking metrics generally had values less than 0.02. Based on Figure 15, we argue that the method proposed in this paper is superior to the application of polynomial fitting, laboratory coefficients and on-orbit coefficients.

Table 2 lists the quantitative values for the images obtained in this paper. It provides the mean values, changes in mean value, RAs, averages of streaking metrics and maximum streaking metrics. The mean values of the raw data were obtained from the non-vignetting areas captured by the two CCDs. The changes in mean value were obtained by comparing the mean values for the corrected images and the raw data. Table 2 shows that the mean value for the images corrected using the laboratory coefficients was larger because the column means in the vignetting area were too large, as shown in Figure 14. The changes in mean value for the images corrected using the proposed method were less than 1%. These observations show that the proposed method yielded the best results among these four methods. The RAs for the images corrected using the proposed method were smaller (<0.1%) than those for the images corrected using the polynomial fitting, laboratory coefficients and on-orbit coefficients reflecting greater uniformity. The averages of streaking metrics for the images corrected using the proposed method were better than 0.02. The maximum streaking metrics values reflect the maximum change in the uniform scene. In the low-brightness region, the maximum difference in streaking metrics between the images corrected using on-orbit coefficients and those corrected using the proposed method exceeded 0.6, whereas the differences in the middle brightness and high-brightness regions were less than 0.1. The maximum streaking metrics of the corrected images using the proposed method were superior to 0.1. The quantitative values shown in Table 2 indicate that the correction effect of the method proposed in this paper is better than those obtained using the other methods.

### 4.3. Accuracy Assessment for the Results from Group C

In this section, three indicators were used: streaking metrics, improvement factor (IF) and energy function of gradient.

To quantitatively evaluate the effect of vignetting correction, in addition to the streaking metrics in Section 4.2, the improvement factor (IF) and energy function (EF) were used to evaluate. To evaluate the correction effect quantitatively, the IF [39,40] was applied to the corrected image. The larger the IF, the better the effect of vignetting correction. It is defined as follows:

(15) $$\;d_{R}\left[i\right]=\;\mu_{IR}\left[i\right]-\;\mu_{I}\left[i\right]\;$$

(16) $$\;d_{E}\left[i\right]=\;\mu_{IE}\left[i\right]-\mu_{I}\left[i\right]\;$$

(17) $$\;IF=10\;\log_{10}\left[\frac{\sum_{i}d_{R}^{2}\left[i\right]}{\sum_{i}d_{E}^{2}\left[i\right]}\right]\;$$

where $\mu_{IR}\left[i\right]$ and $\mu_{IE}\left[i\right]$ are the mean values from the i-th detector in the raw and corrected images, respectively, and $\mu_{I}\left[i\right]$ is the curve obtained by low-pass filtering of $\mu_{IE}\left[i\right]$. Larger IF values reflect higher image quality.

The sharper the image, the better the vignetting correction method. To evaluate the sharpness of the corrected image, the energy function of gradient (EF) [41] was selected to evaluate the sharpness. It is defined as follows:

(18) $$\;EF=\sqrt{\overset{N-1}{\underset{j=1}{\sum }}\overset{M-1}{\underset{i=1}{\sum }}\left({\left|f\left(i+1,j\right)-f\left(i,j\right)\right|}^{2}+{\left|f\left(i,j+1\right)-f\left(i,j\right)\right|}^{2}\right)/\left(M*N\right)}\;$$

where *f* stands for the corrected image, *M* is the number of rows and *N* is the number of columns. Larger EF values reflect higher image quality.

To verify that the proposed method also performed well with images taken in the classic push-broom viewing mode, four types of image data were chosen for verification experiments: images of water, city, hill and desert scenes. The visual effects and quantitative indices were analyzed with images corrected using the laboratory coefficients, the on-orbit coefficients, the polynomial fitting and the proposed method.

**Figure 16:**
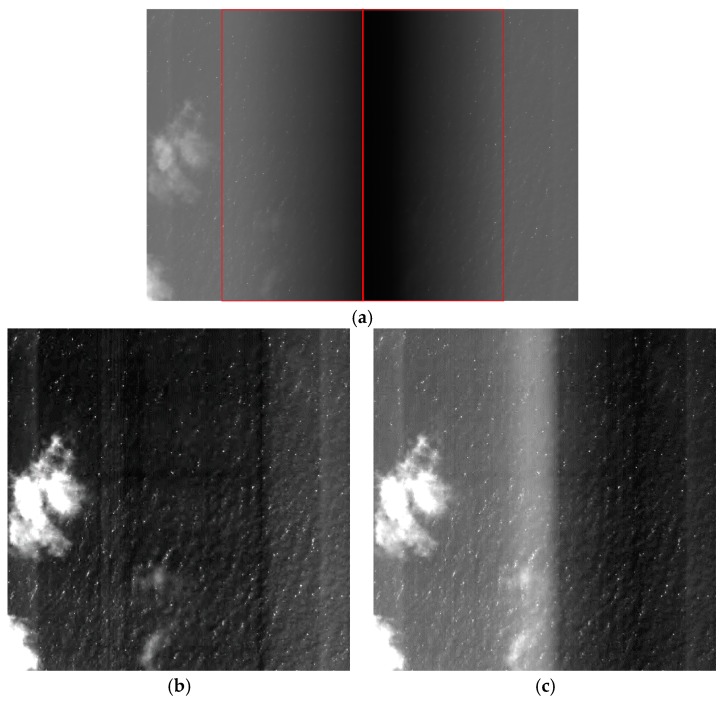

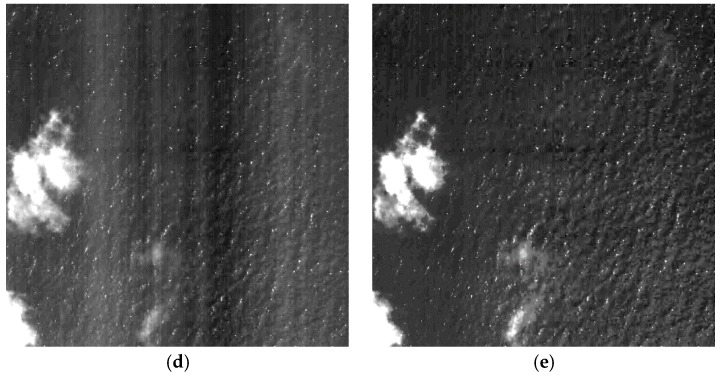
Raw and corrected images of water (the images are stretched for display): (**a**) raw image; (**b**) image corrected using the polynomial fitting; (**c**) image corrected using the laboratory coefficients; (**d**) image corrected using the on-orbit coefficients; (**e**) image corrected using the proposed method.

Figure 16 shows the raw and corrected images of water. Figure 16a shows the raw image produced by two CCDs. Figure 16b–e shows the results of correction using the polynomial fitting, the laboratory coefficients, the on-orbit coefficients and the proposed method, respectively. Figure 16b shows the result of single correction using the polynomial fitting. It shows the non-uniformity between the sides and the middle of the result. Figure 16c shows inconsistencies in gray between the two CCDs, and greater brightness of the vignetting area relative to the other areas, as water is a low-brightness and non-uniform target. The difference in gray between CCDs compromised the correction effect of the laboratory coefficients for the low-brightness target. Figure 16d also shows non-uniformity, indicating that the effect of the on-orbit coefficients was not as expected for the low-brightness target. As can be seen in Figure 16c, the difference between the two CCDs was corrected by the method proposed in this paper. Based on comparison with the vignetting areas in Figure 16b–d, the proposed method showed good performance in vignetting correction (Figure 16e). Thus, the results of the proposed method were superior to the results of using the polynomial fitting, the laboratory or on-orbit coefficients for the water image.

**Figure 17:**
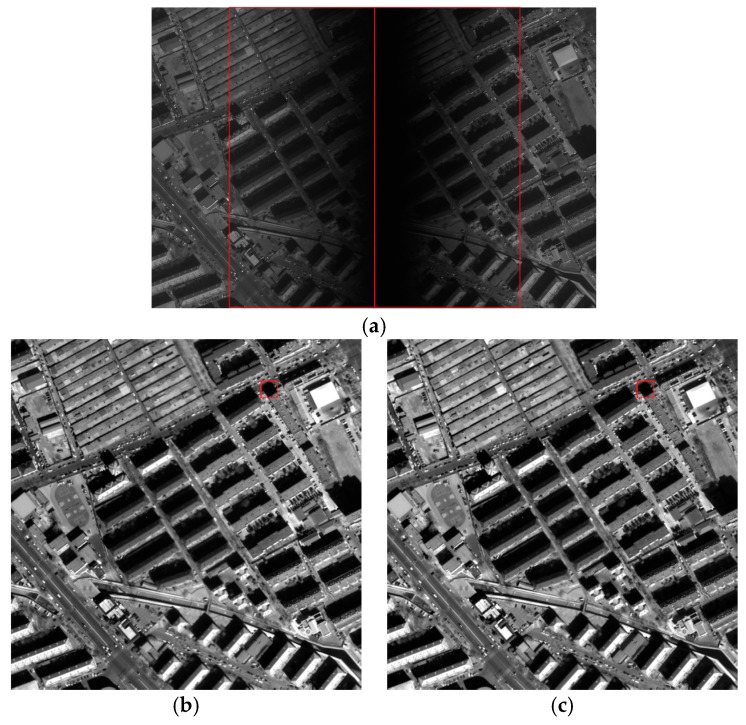

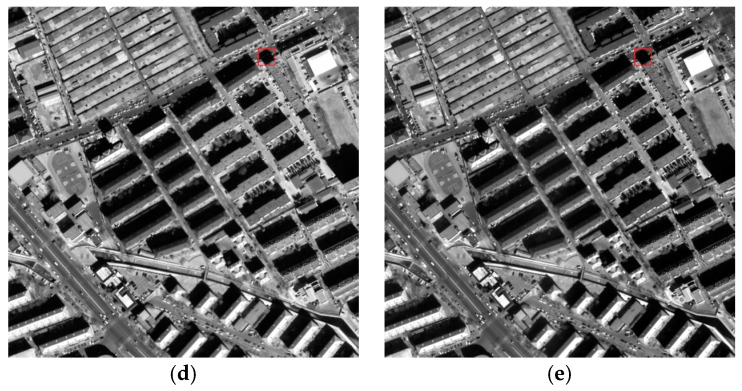
Raw and corrected images of the city (the images were stretched for display): (**a**) raw image; (**b**) image corrected using the polynomial fitting; (**c**) image corrected using the laboratory coefficients; (**d**) image corrected using the on-orbit coefficients; (**e**) image corrected using the proposed method.

Figure 17 shows the raw and corrected images of the city. Figure 17a shows the raw image produced by two CCDs. Figure 17b–d shows the results of correction using the laboratory coefficients, the on-orbit coefficients and the proposed method, respectively. Analysis of the visual effects in Figure 17 revealed no difference among methods in correction of the city image. These three methods performed well in correcting the vignetting area on the raw image of the city. As shown in Figure 17, the information from the vignetting area was completely corrected, which aided subsequent interpretation and the fulfillment of other requirements.

**Figure 18:**
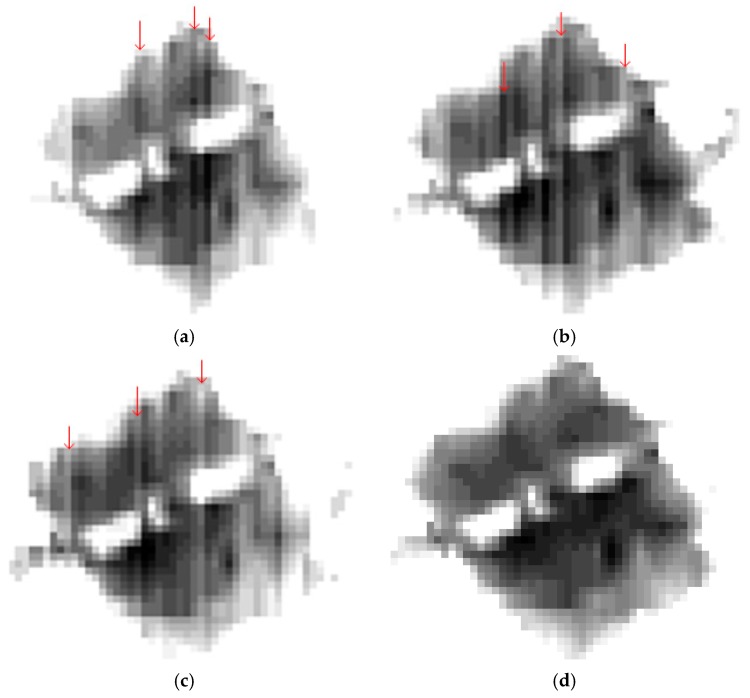
The detail of results (the images were stretched for display). (**a**) Detailed image of the red box showed in Figure 17b; (**b**) detailed image of the red box showed in Figure 17c; (**c**) detailed image of the red box showed in Figure 17d; (**d**) detailed image of the red box showed in Figure 17e.

There are a large number of shadowed areas in the city image as shown in Figure 17. These shadowed areas belong to the low brightness region. Figure 18 shows the details of the red box shown in Figure 17, and the shadowed areas are stretched. Figure 18 shows the details of the four methods in the low-luminance region of the shadow, where the red arrow indicates that the column belongs to the streaking, as can be seen from Figure 18a–c, while the shadowed area in Figure 18d has no streaking. It can be seen that the proposed method performs better in the correction of low brightness, even though the corrected images of using the four methods seem uniform.

**Figure 19:**
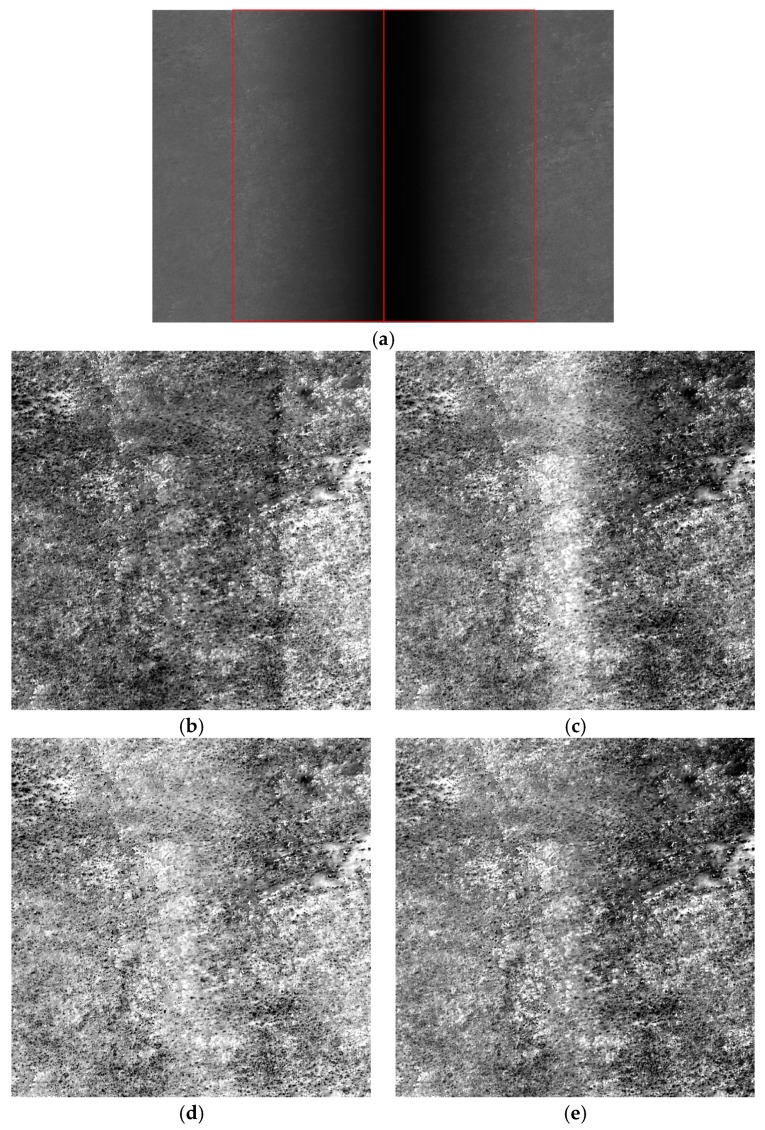
Raw and corrected images of the hill (the images were stretched for display): (**a**) raw image; (**b**) image corrected using the polynomial fitting; (**c**) image corrected using the laboratory coefficients; (**d**) image corrected using the on-orbit coefficients; (**e**) image corrected using the proposed method.

Figure 19 shows the raw and corrected images of the hill. In comparison with the other methods, Figure 19b shows streaking. Figure 19c shows that although the image corrected using the laboratory coefficients exhibited no obvious gray difference, the target was brighter in the corrected vignetting area than in the other areas. Figure 19d shows that the proposed correction across the vignetting region was better than Figure 19c. As can be seen in Figure 19e, the vignetting area did not show high DN, similar to Figure 19d, after the vignetting area was corrected by the proposed method, thus ensuring consistency in features between the vignetting and non-vignetting areas. These results indicate that the correction effect of the proposed method in the vignetting region was better than that obtained using the method based on the polynomial fitting, laboratory coefficients and on-orbit coefficients for the hill image.

**Figure 20:**
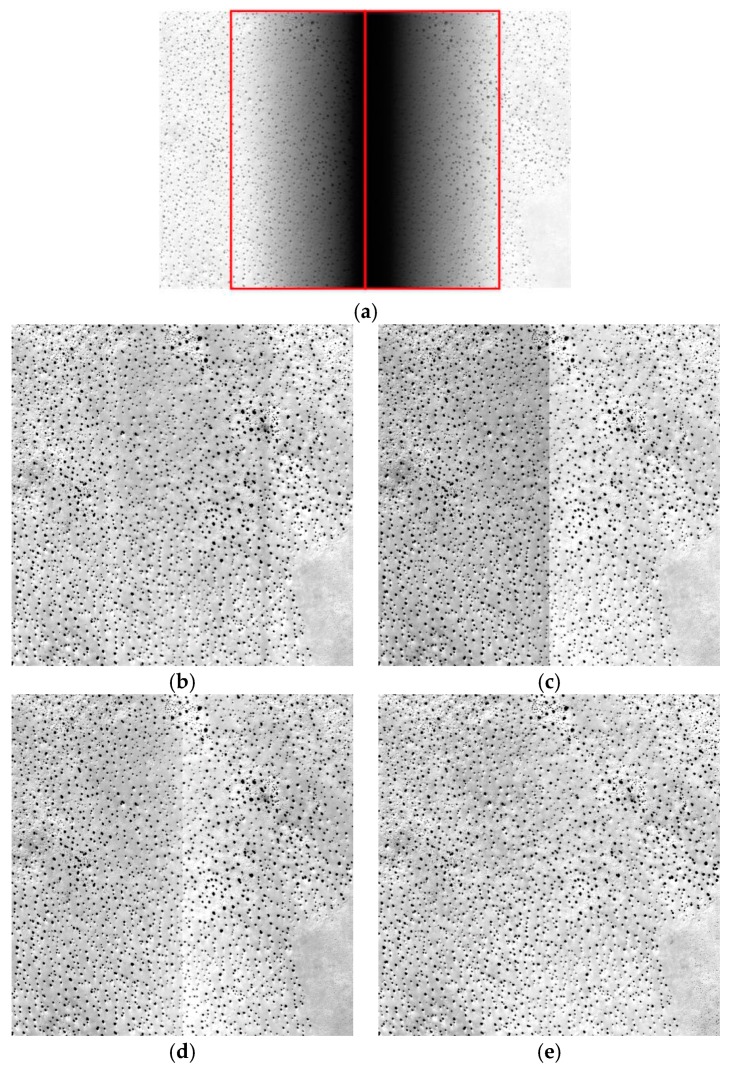
Raw and corrected images of the desert (the images were stretched for display): (**a**) raw image; (**b**) image corrected using the polynomial fitting; (**c**) image corrected using the laboratory coefficients; (**d**) image corrected using the on-orbit coefficients; (**e**) image corrected using the proposed method.

Figure 20 shows the raw and corrected images of the desert. Figure 20a shows the raw image produced by two CCDs. Figure 20b shows the streaking. Figure 20c,d shows the results of correction using the laboratory coefficients and the on-orbit coefficients, respectively. Figure 20c,d shows that the effects of correction were not satisfactory, while Figure 20e shows a better result. Visual analysis of the water, city, hill and desert images indicated that correction using the laboratory coefficients and the on-orbit coefficients was not ideal in areas with less texture, such as those depicting water and desert scenes. However, correction was effective for targets with more texture, such as the city (Figure 17). Compared with the effect of correction using the laboratory coefficients and the on-orbit coefficients, the proposed method performed well for all four types of subject images. The on-orbit coefficients did not perform well on the water image and the desert image. The proposed method showed better performance than the method based on the laboratory coefficients and on-orbit coefficients and improved the uniformity of the images and the quality of the vignetting areas. To analyze the correction effect better, quantitative analyses of the corrected images were performed.

**Figure 21:**
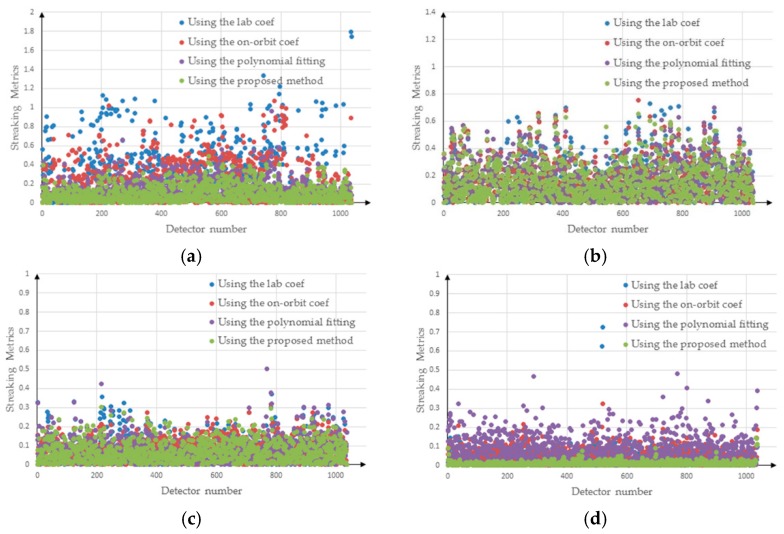
Streaking metrics for the corrected images: (**a**) water; (**b**) city; (**c**) hill; (**d**) desert.

Figure 21 shows the distribution of streaking metrics for images corrected by the laboratory coefficients, the on-orbit coefficients, polynomial fitting and the proposed method. The abscissa represents the detector number, and the ordinate represents the streaking metrics, where the blue dots represent the results obtained using laboratory coefficients, red dots represent the results obtained using on-orbit coefficients, purple dots represent the results obtained using the polynomial fitting and green dots represent the results obtained using the proposed method. Smaller streaking metrics values reflect greater image uniformity. Figure 21a shows the distribution of streaking metrics for the water image. The streaking metrics for the results obtained using the laboratory coefficients were distributed mainly between zero and one, and those for the image corrected by the proposed method were distributed mainly between zero and 0.4. Figure 21b shows the distribution of streaking metrics for the city image corrected by the other coefficients and the proposed method. The distributions ranged from 0–0.8 for both methods. Figure 21c shows the distribution of streaking metrics for the hill image. The streaking metrics between the 200th-detector and 400th-detector were generally greater for the image corrected using the other coefficients than for that corrected using the proposed method. Figure 21d presents the distribution of streaking metrics for the desert image corrected by these four methods. The streaking metrics were distributed roughly between zero and 0.3; while the streaking metrics of the image corrected using the proposed method were generally below 0.1, which is obviously superior to the other three coefficients. For the desert image, the streaking metrics of the corrected image using the polynomial fitting were largest. These results indicate that the proposed method is superior to the methods using the laboratory coefficients, the on-orbit coefficients and the polynomial fitting, such as water and desert. For the city images, the streaking metrics do not differ markedly between correction using the other methods and the proposed method. However, the maximum streaking metrics values are significantly greater for the images corrected using the other two coefficients than for those corrected using the proposed method.

Table 3 lists quantitative values for four types of ground scenes before and after correction. Due to the effect of the vignetting area in the raw data, the means for the raw data in Table 3 were calculated for the non-vignetting area. The changes in mean values for the water, hill and desert images corrected by the proposed method were small, whereas those for the city image were quite large. These results reflect calculation of the raw data only for the non-vignetting area, with the greater change for the city image attributable to the complexity of targets in the vignetting area. For uniform scenes, such as water, hill and desert, the mean values for the images corrected using the laboratory coefficients were much larger than those for images corrected using the proposed method (Table 3). The laboratory coefficients changed the brightness in the vignetting area and improved the brightness of uniform areas, resulting in larger mean values for the corrected images. These results are consistent with the observed visual effect. Higher IF values reflect greater improvement of image quality. Based on IF values, the proposed method showed better performance in improving image quality than did the method using the other three methods for these four images, with obvious improvement in the low-brightness region and the high-brightness region, such as the water and desert images. The average values of streaking metrics for images corrected using the proposed method were less than 0.1 for uniform scenes, such as water, hill and desert and better than the results obtained using other coefficients for the complex target of the city (Table 3). The differences in maximum streaking metrics between the images corrected using the other two coefficients and the proposed method exceeded 0.2 for the water image and were less than 0.15 for the city and hill images. The energy function of the gradient reflects the sharpness of the image, and the edge determines the sharpness of the image. It can be seen from the value of the energy function of these four scenes that the edge information of the city image was more; therefore, the energy function of the city image was higher. The values of the energy function of the proposed method were larger than the other three methods, so the corrected images using the proposed method had a sharper resolution than the other images using that of three methods. These quantitative results indicate that the proposed method performs better than the other methods.

## 5. Discussion

Two sets of experimental data were used to verify the effects of coefficients, and three sets of quantitative indicators were used to evaluate the experimental data. The RA is used mainly to evaluate the field uniformity. The two sets of data verified in this study were the side-slither data and the images obtained using the classical normal push-broom mode. Due to the characteristics of the side-slither data, they can be considered as uniform scenes in the row direction; thus, the RA indicator and the changes in means were used mainly to describe the results of side-slither data. These results demonstrate greater uniformity of the images corrected by the proposed method, with brighter vignetting areas and non-uniformity in low-brightness areas on images corrected using other coefficients. The RA values for the images corrected by the proposed method were 0.0588%, 0.0361% and 0.0334% for the low-brightness, middle brightness and high-brightness areas, respectively, indicating better performance than the other methods. From the viewpoint of changes in mean values, the method proposed here maintained the effect of the mean well, with changes of less than 0.5%. The streaking metrics for low-brightness areas were much lower for the proposed method than for the other methods. Therefore, the proposed method showed superior performance to the other methods with regard to streak removal. The average of streaking metrics for the images corrected by the proposed method was less than 0.02. The differences in maximum streaking metrics between the images corrected with the other methods and those corrected with the proposed method exceeded 0.2 for regions of low brightness. The maximum streaking metrics reflecting the effect of the proposed method were better than for the other methods in low-brightness areas.

With the analysis of the correction results of the images in Group C, it can be seen that the mean values of water, city, hill and deserts are on the rise, from low-luminance areas to high-brightness areas. The correction effects of the four scenes show that the correction effect of using the laboratory coefficient was better on the city image, and the correction effects of using the on-orbit coefficient were better on the city and the hill image, while the correction effects of using the proposed method performed well on these four scenes. The laboratory coefficients were obtained before the satellite was launched, and the coefficients were not effective while the satellite was on-orbit. The on-orbit coefficients used in this paper were calculated using three months of statistical data; the correction effects of the on-orbits coefficient depends on the coverage of the statistical data; and the statistical data will have more mid-brightness areas than low-brightness and high-brightness. Therefore, the corrected images using the on-orbit coefficients were not effective on low-brightness and high-brightness areas. From the results of the vignetting correction using the polynomial fitting, the effect on the city image was better, and the other scenes can be clearly found to have streaking. This is because the polynomial fitting only considers the vignetting function and does not fully consider the influence of the radiometric calibration. In comparison with the proposed method, the polynomial fitting was inferior in sharpness. In this paper, the side-slither data were used for correction. The side-slither data can be used as the uniform field. The power-law model was adopted, and the correction on the low-brightness was considered. The correction effects show that the proposed method was better on the different brightness regions.

## 6. Conclusions

This paper proposes side-slither data-based vignetting correction of a high-resolution spaceborne camera with an optical focal plane assembly for the vignetting problem. In this paper, the side-slither data had been standardized, the sample points were acquired and the coefficients based on the power-law model were calculated. In the experiment, three methods of polynomial fitting, laboratory coefficients and on-orbit coefficients were compared. This paper mainly evaluates the corrected images from both qualitative and quantitative aspects. The Group B data can be regarded as the uniform scene, in which the RA and the streaking metrics were used to evaluate the correction effect of the uniform scene. It can be seen from the visual effect that the correction results of the other three methods appear non-uniform on the low-brightness region. For the middle brightness and high-brightness areas, for the polynomial fitting method, obvious streaking can be seen. From the visual effects of these three different brightness areas, the proposed method is obviously superior to the other three methods. With the quantitative analysis of the Group B data, the RA values for these three regions using the proposed method are 0.0588%, 0.0361%, and 0.0334%, respectively. The maximum streaking metrics were lower than 0.1, which is far superior to other methods. Therefore, the proposed method is better than the other methods of removing the non-uniformity of the scene. The Group C data include four different scenes of water, city, hill and desert. From the visual effect analysis, these four methods a have good correction effect for the city, but after zooming into the shadow of the city, it can be seen that the proposed method is obviously better than the other three methods. The results of the water scene show that the effect using the proposed method was better. Table 3 shows that the proposed method was superior to the other three methods in terms of quality improvement and sharpness improvement. Therefore, we conclude that the proposed method is superior to those using the other three methods.

## Figures and Tables

**Table 1:** Details of the experimental data.

Group Name	Object Type	Imaging Mode	Uses	Imaging Time
Group A	Side-slither data	Side-slither scan	Calibration	3 January 2015
Group B	Side-slither data	Side-slither scan	Verification	16 March 2015
Group C	Water	Classical	Verification	27 February 2015
	City	Classical	Verification	24 January 2015
	Hill	Classical	Verification	13 April 2015
	Desert	Classical	Verification	21 March 2015

**Table 2:** Comparison of quantitative values for the images.

Region	Correction Method	Mean Value (MV)	Changes in MV (%)	RA (%)	Average of Streaking Metrics	Maximum Streaking Metrics
Low-brightness	Raw data (non-vignetting area)	152.6908	/	/	/	/
	Laboratory coefficients	155.3894	1.7674	4.8410	0.1267	0.7451
	On-orbit coefficients	157.0680	2.8667	2.5901	0.2236	0.7771
	polynomial fitting	157.7402	3.3069	0.0971	0.0181	0.7082
	Proposed method	152.3096	−0.2496	0.0588	0.0163	0.0810
Middle brightness	Raw data (non-vignetting area)	380.5334	/	/	/	/
	Laboratory coefficients	384.6584	1.0840	1.5108	0.0291	0.3089
	On-orbit coefficients	385.6701	1.3499	0.8300	0.0373	0.1003
	polynomial fitting	387.2508	1.7653	0.0519	0.0152	0.2815
	Proposed method	381.9701	0.3775	0.0361	0.0066	0.0365
High-brightness	Raw data (non-vignetting area)	672.4643	/	/	/	/
	Laboratory coefficients	676.9013	0.6598	1.6432	0.0275	0.3593
	On-orbit coefficients	669.9590	−0.3726	0.6241	0.0155	0.0994
	polynomial fitting	679.6329	1.0660	0.0439	0.0073	0.4815
	Proposed method	672.8545	0.0580	0.0334	0.0022	0.0131

**Table 3:** Comparison of quantitative values for the images. IF, improvement factor.

Region	Correction Method	Mean Value (MV)	Changes in MV (%)	IF	Average of Streaking Metrics	Maximum Streaking Metrics	Energy Function
Water	Raw data (non-vignetting area)	105.7267	/	/	/	/	3.0580
	Laboratory coefficients	117.1819	10.8348	14.3114	0.2458	1.7952	3.5212
	On-orbit coefficients	109.9966	4.0386	16.0074	0.1946	1.0696	3.4602
	polynomial fitting	118.5100	12.0909	20.2544	0.1485	0.9639	3.1627
	Proposed method	107.0849	1.2846	23.8594	0.0993	0.4933	3.5878
City	Raw data (non-vignetting area)	269.2928	/	/	/	/	27.3414
	Laboratory coefficients	267.9996	−0.4802	12.9659	0.1782	0.7290	32.7385
	On-orbit coefficients	261.6164	−2.8506	11.5552	0.1627	0.7549	31.1614
	polynomial fitting	290.2502	7.7824	10.7418	0.1694	0.6984	31.4548
	Proposed method	260.9379	−3.1025	12.6999	0.1622	0.6545	33.0428
Hill	Raw data (non-vignetting area)	374.2867	/	/	/	/	9.9862
	Laboratory coefficients	382.8244	2.2811	21.0964	0.0796	0.3706	10.9966
	On-orbit coefficients	376.2886	0.5349	27.1364	0.0793	0.3167	13.3237
	polynomial fitting	378.1881	1.0424	24.0872	0.0710	0.5042	11.3148
	Proposed method	377.4831	0.8534	27.6778	0.0701	0.3029	13.4969
Desert	Raw data (non-vignetting area)	675.8800	/	/	/	/	11.1431
	Laboratory coefficients	662.9942	−1.9065	36.8014	0.0379	0.7256	13.3153
	On-orbit coefficients	679.8685	0.5901	39.4644	0.0464	0.3239	13.5696
	polynomial fitting	673.6525	0.3296	44.0960	0.0835	0.4801	12.0047
	Proposed method	675.4349	−0.0659	50.6899	0.0113	0.1467	15.4989
